# Photocatalytic degradation of Congo red dye using innovative cerium titanate nanorods embedded in a cellulose-based hydrogel

**DOI:** 10.1038/s41598-026-43425-8

**Published:** 2026-04-16

**Authors:** Ahmed M. Khalil, Samir Kamel, Mohamed S. Mohy-Eldin

**Affiliations:** 1 Photochemistry Department, National Research Centre, Dokki, Giza, 12622 Egypt; 2 Cellulose and Paper Department, National Research Centre, Dokki, Giza, 12622 Egypt; 3 Polymer Materials Research Department, Advanced Technology and New Materials Research Institute (ATNMRI), City of Scientific Research and Technological Applications (SRTA-City), New Borg Al Arab, Alexandria, 21934 Egypt

**Keywords:** Congo red, Adsorption, Photo-degradation, Photo-catalyst, Carboxymethyl cellulose/polyacrylamide, Cerium titanate nano-rods, Composite hydrogel, Chemistry, Environmental sciences, Materials science, Nanoscience and technology

## Abstract

This work used a simple technique to prepare a novel cerium titanate nano-rods embedded in a cellulose-based hydrogel with high photo catalytic degradation activity. The composite hydrogel was prepared by embedding different amounts of cerium titanate nano-rods (Ce/Ti-NRs) into a crosslinked carboxymethyl cellulose/polyacrylamide polyelectrolyte complex (CMC/PAM) as a matrix material. The physico-chemical properties of the Ce/Ti-NRs, CMC/PAM, and cerium titanate nano-rods embedded in a cellulose-based hydrogel (Ce/Ti-NRs/CMC/PAM) were investigated by FT-IR, XRD, BET, TEM, SEM, and EDX techniques. The photo-degradation efficiency of the prepared composite hydrogel was investigated for their ability to simultaneously adsorb-degrade Congo red dye (CR) under different conditions such as the dosage of composite hydrogel, dye solution pH and temperature, degradation time, initial dye concentration, and agitation rate. Also, the kinetics of the CR degradation process was evaluated. The obtained data fits well using pseudo-first order kinetic model with R^2^ equal to 0.911 and calculated equilibrium capacity value (22.44 mg/g) closer to the corresponding experimental values (23.193 mg/g) than those of pseudo-second order model with R^2^ equal to 0.888 and calculated equilibrium capacity value (34.78 mg/g). The linear plot of the intra-particle model indicated that the simultaneously adsorb-degrade of CR dye by the composite hydrogel is likely to be complex. It involves both film diffusion (boundary layer diffusion) and intra-particle diffusion. The optimum developed composite hydrogel shows high simultaneously adsorb-degrade photo catalytic activity superior to other published results as it degraded 91.68% of CR dye only after 90 min.

## Introduction

Industrial water contamination is a significant global issue. Dyes from the textile industry contaminate the environment due to their vivid pigmentation and carcinogenic properties. Approximately 1–20% of the entire global dye production from textile companies is lost during the dyeing and printing processes^1^. This leads to eutrophication and disrupts living organisms^2–4^. Consequently, the elimination of colors from wastewater has been thoroughly addressed in the past decade. Numerous physical, chemical, and biological techniques, including adsorption onto activated carbon, coagulation, flocculation, chlorination, ozonization, and biodegradation, are the predominant methods employed for the removal of textile dyes from wastewater^5–8^. Although these approaches are non-destructive, they only shift pollutants from one phase to another, resulting in secondary pollution that necessitates additional treatment. Likewise, biological approaches are ineffective for dye removal because colors adhere to the biomass surface rather than undergoing breakdown and de-colorization. To address these challenges, a highly efficient and environmentally friendly advanced oxidation approach, specifically heterogeneous photo catalysis utilizing semiconductor materials, has emerged as the most appropriate method for the removal of textile dyes from wastewater^9–12^. Titanium dioxide (TiO_2_) is a highly promising and extensively utilized semiconductor material for environmental remediation and energy applications. This is attributed to its high reactivity, chemical stability, affordability, non-toxicity, and resilience under irradiation^13–16^. Traditionally, TiO_2_ nanoparticles (TiO_2_-NPs) have been employed in wastewater treatment owing to their extensive surface area facilitating photo catalytic degradation^17,18^. Nevertheless, dispersed TiO_2_ nanoparticles are challenging to retrieve, resulting in secondary contamination. Consequently, TiO_2_ nanoparticles can be immobilized on many substrates, including glass, stainless steel, aluminum, activated carbon, silica, carbon nanotubes, and polymers^16,19–25^. Li et al. have effectively created an innovative double-cylindrical-shell (DCS) photo reactor, immobilized with monolayer TiO_2_-coated silica gel beads, and successfully utilized it for the degradation of rhodamine B and methyl orange^26^. Recently, TiO_2_ nano-particles were encapsulated within a polyvinyl alcohol (PVA) matrix using a solution-casting technique followed by heat treatment^18^. Polysaccharide-based matrices, including cellulose, chitosan, and alginate, have been employed for the immobilization of TiO_2_ nano-particles to improve their applications and properties, owing to some drawbacks such as inadequate matrix compatibility, exuviation of TiO_2_ from the matrix, and suboptimal pliability^27–29^. Molly Thomas et al. described the production and characterization of TiO_2_ nano-particles encased in alginate-carboxymethyl cellulose (CMC) gels through the crosslinking of their solutions with barium ions, utilizing a dissipative convective phenomenon, followed by a freeze-drying approach. The produced Ba/Alg/CMC/TiO_2_ gels have been utilized for the photo catalytic degradation of Congo red dye (CR)^30^. Recently, Sugashini et al. conducted a study in which chitosan biopolymer, in the form of nano-chitosan (NCS), and carboxymethyl cellulose (CMC) were utilized as support materials for TiO_2_ to address its limitations. These materials are essential for augmenting the active surface area, optimizing dye degradation efficacy, and bolstering the chemical stability of the composite catalyst. The adsorption capacities of NCS and CMC facilitate dye degradation by positioning dye molecules near the active sites of TiO_2_, where hydroxyl radicals are produced. Moreover, holes and electrons efficiently interact with dye species adsorbed on the TiO_2_ catalyst surface to facilitate efficient dye degradation^31^. Currently, the photo catalysis of titanium dioxide under visible light has generated significant interest in utilizing sunlight. The metallization of TiO_2_ has enabled the synthesis of photo catalysts active under visible light^32^. Despite numerous applications of meso-porous TiO_2_ powder and films reported to date, two significant deficiencies impede catalyst efficiency: (i) its broad energy spectrum permits activation solely in the ultraviolet region (approximately 3–5% of the total solar spectrum) and (ii) the swift recombination of photo generated charges. An effective approach to mitigate the second shortfall in TiO_2_ photo catalytic activity is to alter TiO_2_ with diverse elements, particularly rare earth metal ions and transition metals^32,33^. Consequently, numerous prior investigations have employed a multifaceted strategy to synthesize cerium titanate (Ce-Ti) nano-particles, nanorods, porous structures, and cerium-doped TiO_2_^34–38^. Recently, Yousra H. Kotpa developed a highly efficient sunlight-responsive photo catalyst utilizing cellulose fibers, CeO_2_ nano-particles, and diverse ratios of Ce-Ti nano-particles synthesized through eco-friendly methods. The Ce-Ti/Cf nano-composite was subsequently synthesized, exhibiting a remarkable degradation efficiency for methylene blue (MB) and methyl orange (MO) dyes. Based on the obtained results, the synthesized photo catalyst from Ce-Ti/Cf particles may serve as an effective material for the treatment of dye wastewater^39^. Aradhana Chaudhary et al.^40^ developed of a biodegradable chitosan–TiO_2_ hydrogel nano-composite that combines both adsorption and photo catalytic degradation capabilities of CR dye within a single, cost-effective material utilized at different temperatures and pH levels. The maximum percentage degradation was found to be 95.44% at 65 °C.

Benefiting from the previous findings^31,39,40^ new, in the current study, a new photo catalyst hydrogel was prepared by embedding different amounts of cerium titanate nano-rods (Ce/Ti-NRs) into carboxymethyl cellulose/polyacrylamide (CMC/PAM) as a matrix material. The nano-rod morphology provides elongated one-dimensional pathways that facilitate directional charge transport and suppress electron–hole recombination, thereby enhancing photo catalytic efficiency compared to conventional nano-particle systems. Simultaneously, the three-dimensional, crosslinked hydrogel network offers a highly porous and hydrated environment that not only immobilizes the nano-rods with excellent stability but also promotes rapid diffusion and adsorption of dye molecules onto active sites. This synergistic integration improved charge separation and enhanced simultaneous adsorption–degradation coupling processes; which together overcome the limitations of fiber-supported or particle-loaded composites. Thus, the Ce/Ti-NRs/CMC/PAM composite hydrogel represents a novel and more effective platform for sustainable dye removal, bridging structural stability with advanced photo catalytic performance.

Congo red (CR), one of the most difficult azo dyes frequently released by the textile industry, was chosen as the study’s target pollutant. Its extensive conjugated system and complex aromatic structure confer strong chemical stability and resistance to biodegradation. However, there are significant concerns to the environment and human health due to its toxicity and possible carcinogenicity. Because of these qualities, CR is both a chronic pollutant in aquatic systems and a strict standard for assessing the effectiveness of new photo catalytic materials.

The photo degradation efficiencies of the prepared photo catalyst composite hydrogel were investigated for their ability to degrade Congo red dye under visible light through study the effect of the photo catalyst concentration in the polymer composite hydrogel, amount of the photo catalyst composite hydrogel, dye solution pH, dye solution temperature, dye degradation time, and initial dye concentration. In addition, the kinetic of the dye removal process were evaluated. Therefore, demonstrating effective degradation of CR provides strong evidence of the robustness of the developed Ce/Ti-NRs/CMC/PAM composite hydrogel.

## Materials and method

### Materials

Carboxymethyl cellulose (CMC), (M.Wt. 90,000, DS = 0.7), was purchased from New Jersey (USA) in the form of sodium salt. Polyacrylamide (PAM) (MW = 150,000 g/mol), was supplied from Sigma Aldrich Company. N,N′-methylene-bis acrylamide, 99% (MBA) as a cross-linker, and ammonium persulfate98% (APS) as an initiator were purchased from Sigma-Aldrich Chemie GmbH, Germany. Titanium dioxide was provided from Finar Chemicals Limited, India. Ceric ammonium sulfate dehydrate (NH_4_)Ce(SO_4_)_3_H_2_O was purchased from Aldrich Chemical Co. Ltd. Sodium hydroxide (NaOH), hydrochloric acid (HCl, 6N), acetic acid, Congo red and were obtained from Fisher Scientific Co. (Norcross, GA, USA. All chemicals were of analytical grade and used without any further purification.

### Preparation of adsorbents

#### Preparation of cerium titanate

3 g of TiO_2_ was dispersed in 90 mL aqueous NaOH (10 mol/L) using magnetic stirring for 30 min. Afterwards, they were transferred into Teflon reactor and subjected to irradiation of microwave for 3 h at 150 °C^41^. The obtained solid product was washed with distilled water and dried in an oven, producing sodium titanate decorated with anatase nano-rods (Na-Ti-NRs). 300 mg of Na-Ti-NRs was suspended in 300 mL of an aqueous solution of Ce (0.05 mol/L) using a magnetic stirrer at room temperature for 24 h, and the pH was adjusted to ~ 5. The solid content was isolated by centrifugation and washed several times with distilled water. The solid product was dried, yielding cerium-ion titanate nano-rods (Ce/Ti-NRs)^42^.

#### Preparation of hydrogel^43^

**Scheme 1:**
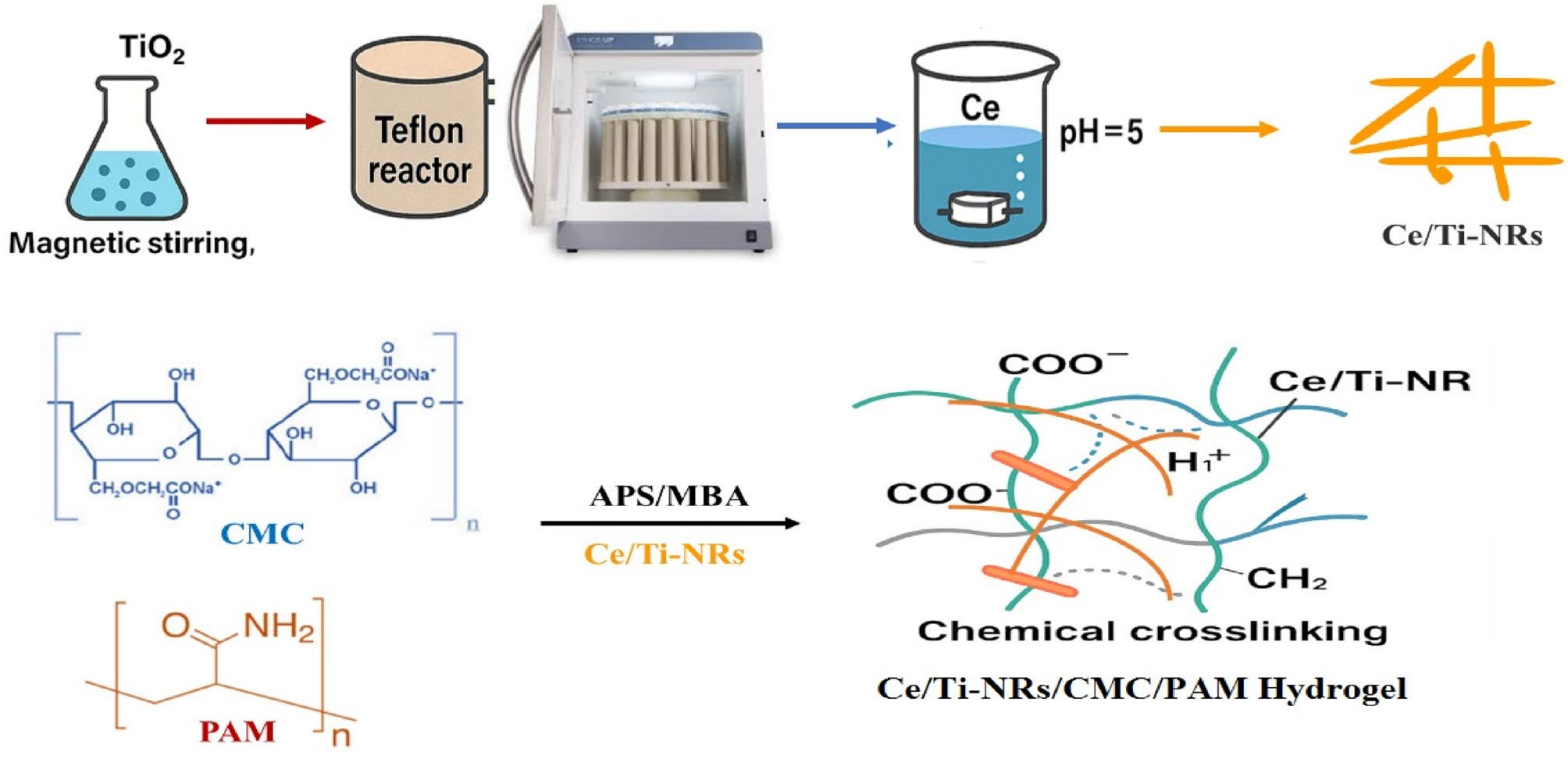
Flowchart of the preparation of Ce/Ti-NRs/CMC/PAM composite hydrogel.

CMC (2 g/100 ml) and PAM (2 g/100 mL) (50/50 wt. %) polymers were dissolved in distilled water for 8 h at room temperature to confirm the complete dissolution for the used polymers. After that, these solutions were mixed for 4 h at room temperature to depict the homogeneity of the blend mixture. The nano-filler powder (Ce/Ti-NRs)(0.05 g/10 mL) was also dispersed in distilled water and sonicated by a probe sonicator (Q500-Sonicator, USA). Then, it was mixed to the solution of CMC/PAM blend with different weight percentages from 0 to 2 wt % according to Table 1, 0.12 g of APS as imitator and 0.12 g MBA were added as a cross-linker with continuous magnetic stirring about 1 h at 55 °C (Scheme 1). After that, it was cast onto Teflon Petri dishes and put in the oven at 60 °C to dry, with grinding to reach size between 400 and 600 microns. The prepared composites were coded as displayed in Table 1.

**Table 1:** The codes of Ce/Ti-NRs/CMC/PAM composite hydrogel (PAC).

Code No	PAC1	PAC2	PAC3	PAC4	PAC5
Ce/Ti-NRs	0.00	0.25	0.5	1	2

### Characterizations

#### Transmission electron microscope (TEM)

TEM images of Ti-NRs and Ce/Ti-NRs were shot with a JEOL JEM-2100 electron microscopy at an acceleration voltage of 120 kV.

#### Fourier transforms infrared (FT-IR) spectra

FT-IR spectra were recorded in the range of 400–4000 cm^-1^on Perkin Elmer Spectrum 100 T FT-IR spectrometer.

#### Scanning electron microscopy with energy dispersive electron spectroscopy (EDX-SEM) surface morphology

The surface morphology was analyzed using FEI Phillips Scanning Electron Microscope (SEM), environmental scanning with coating. The spatial elemental distribution of the samples was studied using the non-destructive energy dispersive X-ray (EDX) unit attached to scanning electron microscopy. Imaging the surface morphology of different samples was recorded using an accelerating voltage of 10–15 kV.

#### X-ray diffraction (XRD)

The XRD patterns were investigated on a Diano X-ray diffractometer using CoKα radiation source energized at 45 kV and a Philips X-ray diffractometer (PW1930 generator, PW 1820 goniometer) with CuK radiation source (λ = 0.15418 nm), at a diffraction angle range of 2θ from 10 to 80° in reflection mode.

#### Nitrogen gas adsorption and surface properties (BET)

The specific surface area, pore volume, and pore size of Ce/Ti-NRs/CMC/PAM composite hydrogel were determined using a specific surface area and porosity analyzer (Micromeritics of USA, TriStar II Plus Series). The sample was pre-heated at 120 °C, followed by gassing at 200 °C under a flow of N2 for 3 h, and then measured in liquid nitrogen. The Barrett-Joyner-Halenda (BJH) method was used to measure pore size and pore volume.

### Dye removal study

In order to assess the dye removal (adsorption and photo degradation) procedure, bulk dye removal experiments were conducted. The desired concentration was attained by dilution, for a stock solution of 1000 ppm CR dye by dissolving 0.1 g of each dye in 100 mL of distilled water. In 25 mL of CR dye solution (100 mg/L), 0.1 g of crosslinked CMC/PAM hydrogel or Ce/Ti-NRs/CMC/PAM composite hydrogel was accurately inserted in an Erlenmeyer flask. The pH of the adsorption medium was adjusted between 2.0 and 8.0 using 0.1 M solutions of both HCl and NaOH for contact time (0–180 min.). Experiments were conducted at a constant rate of agitation (100 rpm) and a temperature of 25 °C. Then, it has reached equilibrium by separating the polymer hydrogel adsorbent from the dye solution via centrifugation at 6000 rpm for 5 min. At an absorbance wavelength of 497 nm, a UV–Vis spectrophotometer was utilized to measure the residual concentration of CR dye.

The isotherms, thermodynamics, and kinetics of dye removal were investigated. The CR dye residual concentration in the aqueous solution was calculated using the initial dye concentration and absorbance measurements before and after adsorption. The dye removal percentage (R%) and dye removal capacities of CR at time t were determined using the following equations:

1 $$R\,\left( \% \right) = \frac{{c_{0} - c_{t} }}{{c_{0} }} \times 100$$

2 $$q_{t} = \frac{{\left( {c_{0} - c_{t} } \right)\upsilon }}{1000w}$$

C_0_ is the initial dye concentration (mgL^-1^), C_t_ is the dye concentration at different time intervals (mgL^-1^), V is the volume of dye solution (L), and W is the mass of the polymer hydrogel matrix (g). R (%) represents the dye removal efficiency of CR dye using the polymer hydrogel matrices, and q_t_ is the dye removal capacity (mg/g). To evaluate the dye-removal adsorption capacity, the Ce/Ti-NRs/CMC/PAM composite hydrogel was used in the absence of visible light. On the other hand, the photo catalytic degradation capacity was evaluated under visible light, using a 300 W xenon lamp equipped with a cut-off filter (λ > 420 nm) at a lamp-to-sample distance of 15 cm. For the sunlight experiment, it was performed outdoors under clear-sky conditions at noon in Cairo, Egypt, during July, with the sample placed at a fixed distance with no shading.

## Results and discussion

A crosslinked CMC/PAM hydrogel has been prepared as a new polymer-based material for the prepared Ce/Ti-NRs/CMC/PAM composite hydrogel to be used as a photo degradation matrix for the CR dye. The crosslinked CMC/PAM hydrogel has two roles. The first role is the attraction of the CR dye by adsorption process from the dye solution. The positive charged adsorption centers are formed by the formation of the CMC/PAM polyelectrolyte through protonation of the PAM amine groups using the hydrogen ions of the CMC carboxylate groups. As a sequence, a physical crosslinking resulted. The second role is the immobilization of the Ce/Ti-NR to have the Ce/Ti-NRs/CMC/PAM composite hydrogel. The stability of the formed hydrogels and preventing the Ce/Ti-NRs leakage from the polymer matrix, a second chemical crosslinked process was performed using N,N′-methylene bisacrylamide as a monomer and persulphate as an initiator Many possibilities here could be considered namely as follow:

- Covalent crosslinking the CMC,
- Covalent linking of the PAM to the CMC,
- Covalent crosslinking the PAM.

### Materials characterization

The synthesized nano-catalyst materials (Ti-NRs and Ce/Ti-NRs) alongside the crosslinked CMC/PAM hydrogel and Ce/Ti-NRs/CMC/PAM hydrogel have been characterized using different characterization analytical techniques; namely TEM, FTIR, XRD, SEM and EDX to verify the chemical structure and the CR dye photo degradation process.

#### TEM

**Fig. 1:**
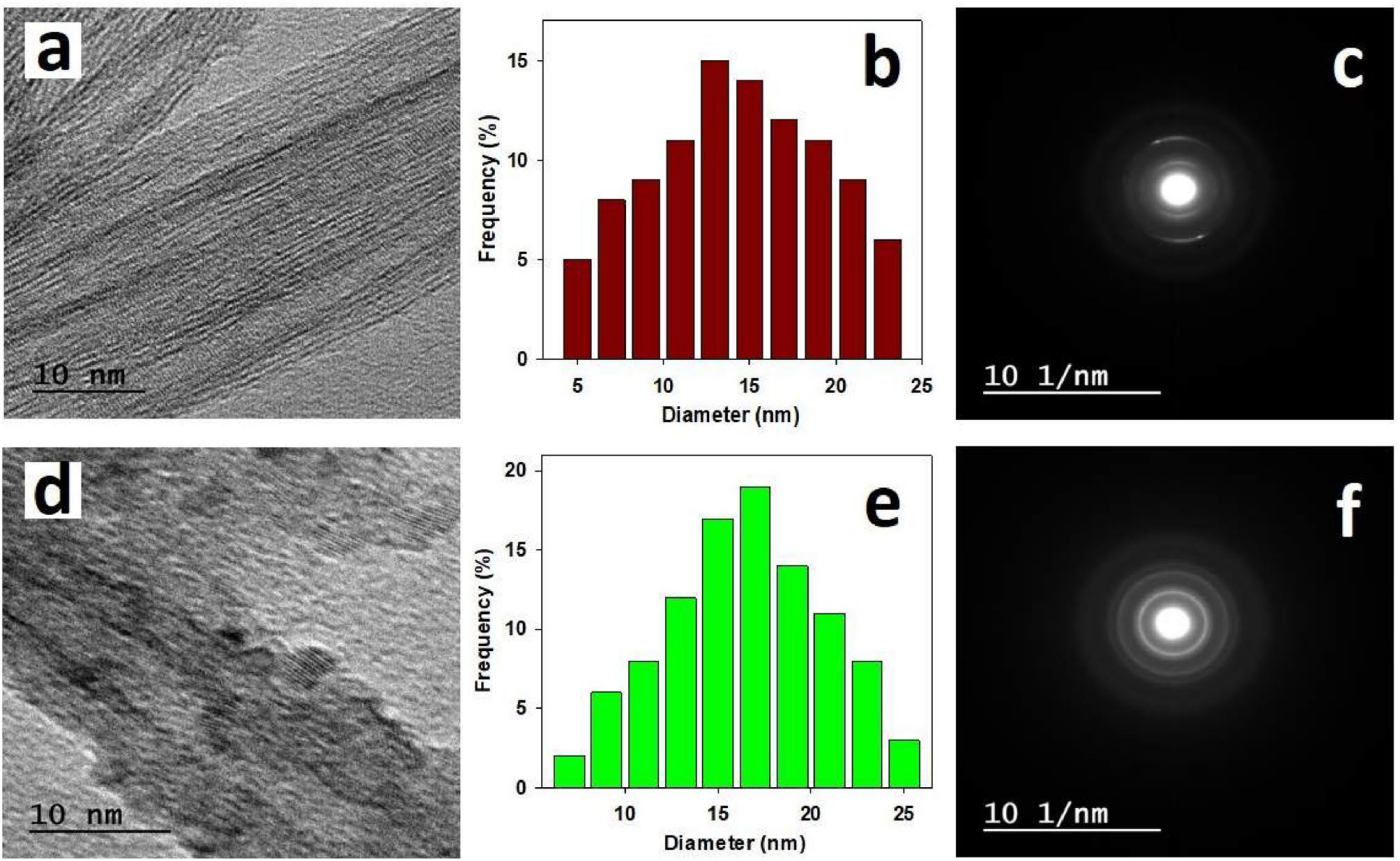
TEM (**a**, **d**), diameter distribution (**b**, **e**), and selected area electron diffraction (SAED) of Ti-NRs and Ce/Ti-NRs (**c**, **f**), respectively.

Figure 1a depicts the TEM micrograph of Ti-NRs with a predominantly extending morphology and minimal agglomeration. The high-resolution TEM image corresponds to an inter planar spacing (d-spacing) of about 0.35 nm characterize the (101) plane of anatase TiO_2_. The clear edges of Ti-NRs confirm their high purity and crystallinity, and the absence of visible amorphous regions suggests a well-ordered crystal structure. They appeared as hollow tubular structures with outer diameters in the range of 8–12 nm and lengths up to several hundred nanometers as shown in Fig. 1b. Figure 1c displays the selected area electron diffraction (SAED) pattern of Ti-NRs, showing a concentric diffraction rings with a series of bright confirming the polycrystalline nature. The d-spacing correspond to the (101), (004), (200), (105), and (204) planes of anatase TiO_2_, in agreement with the standard JCPDS card No. 21–1272. The absence of diffuse halos confirms the minimal presence of amorphous phases, phase-pure anatase Ti-NRs. In Fig. 1d, the TEM image of the Ce/Ti-NRs illustrates the formation of tubular nanostructures decorated with well-dispersed nano-rods. The anchored nano-particles are attributed to Ce, which appear as darker contrast spots uniformly distributed along the Ti-NRs surfaces. This morphology suggests successful deposition of Ce nano-particles onto the Ti-NRS without significant aggregation. The intimate interfacial contact between Ti-NRs and Ce nano-particles is expected to facilitate efficient charge transfer between components, enhancing the photo catalytic and redox performance. This image revealed distinct lattice fringes corresponding to both TiO_2_ (d = 0.35 nm for the (101) plane) and CeO_2_ (d = 0.31 nm for the (111) plane), confirming the coexistence of the two crystalline phases within the hybrid nanostructure. Moreover, the size of the developed nanostructure shows an increase as plotted in Fig. 1e with diameter in the range of 18 nm exceeding that of the reference Ti-NRs. The SAED pattern of the Ce/Ti-NRs (Fig. 1f) exhibited multiple diffraction rings corresponding to the anatase phase of TiO_2_ and the fluorite structure of CeO_2_. The indexed rings matched the (101), (200), and (211) planes of anatase TiO_2_ and the (111), (200), and (220) planes of CeO_2_, in agreement with JCPDS No. 21-1272 and 34-0394, respectively. The presence of discrete diffraction spots superimposed on the rings suggests partial orientation of the CeO_2_ nano-particles along the Ti-NRs framework. The overall intensity and sharpness of the rings confirm high crystallinity of the hybrid system. The coexistence of TiO_2_ and CeO_2_ reflects without any additional unidentified peaks successful incorporation of Ce and Ti nano-particles without structural distortion or formation of secondary phases.

#### FT-IR

**Fig. 2:**
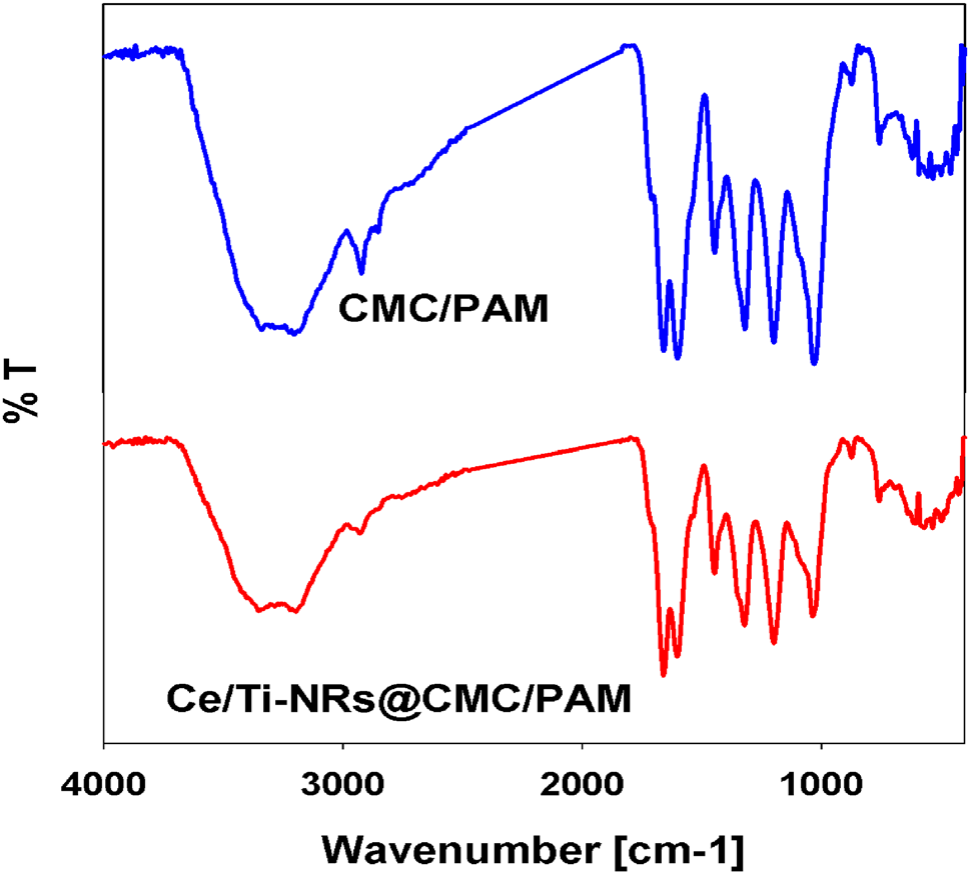
FTIR spectra of CMC/PAM hydrogel and Ce/Ti-NRs/CMC/PAM composite hydrogel.

The FT-IR spectra of the crosslinked CMC/PAM hydrogel and Ce/Ti-NRs/CMC/PAM composite hydrogel are shown in Fig. 2. From the spectrum of CMC/PAM hydrogel, it showed a broad absorption band around 3440 cm^-1^, due to the stretching frequency of the CMC-OH groups and the N–H stretching frequency of the NH_2_ group. The band at 2921 cm^-1^ was due to C-H stretching vibration. Appearance of a strong absorption band at 1600 cm^-1^ was due to the presence of COO- groups. The bands around 1444 and1318 cm^-1^ were assigned to CH_2_ scissoring and-OH bending vibration of CMC and CH_2_ scissoring andCH_2_ twisting of PAM, respectively. Two bands around 1689 and 1657 cm^-1^ were due to amide-I (C = O stretching) and amide-II (NH bending), respectively^44–46^. Also, there was an important peak at 1028 cm^-1^which is assigned for the CH-O-CH_2_ group resulting from crosslinking reaction between the hydroxyl groups located in anhydro-glucose C2 position and the p-bond of the bisacrylamide cross-linker. The primary peaks existed in the CMC/PAM hydrogel characteristic for the groups of acrylamide. The shift in the band corresponding to OH group, may suggest formation of ether (-CH-O-CH_2_) during the cross-linking copolymerization. Accordingly, it is apparent that FTIR presented strong evidence of crosslinking the polysaccharide backbone.

In addition to the characteristic bands of the CMC/PAM hydrogel, the IR spectrum of Ce/Ti-NRs/CMC/PAM composite hydrogel shows bending vibration of the Ti–O bond has a wide absorption peak around 660 cm^-1^ and light absorption around 530 cm^-1^ was because of the vibration of the Ti–O-Ti vibration stretching^39^.

#### XRD

**Fig. 3:**
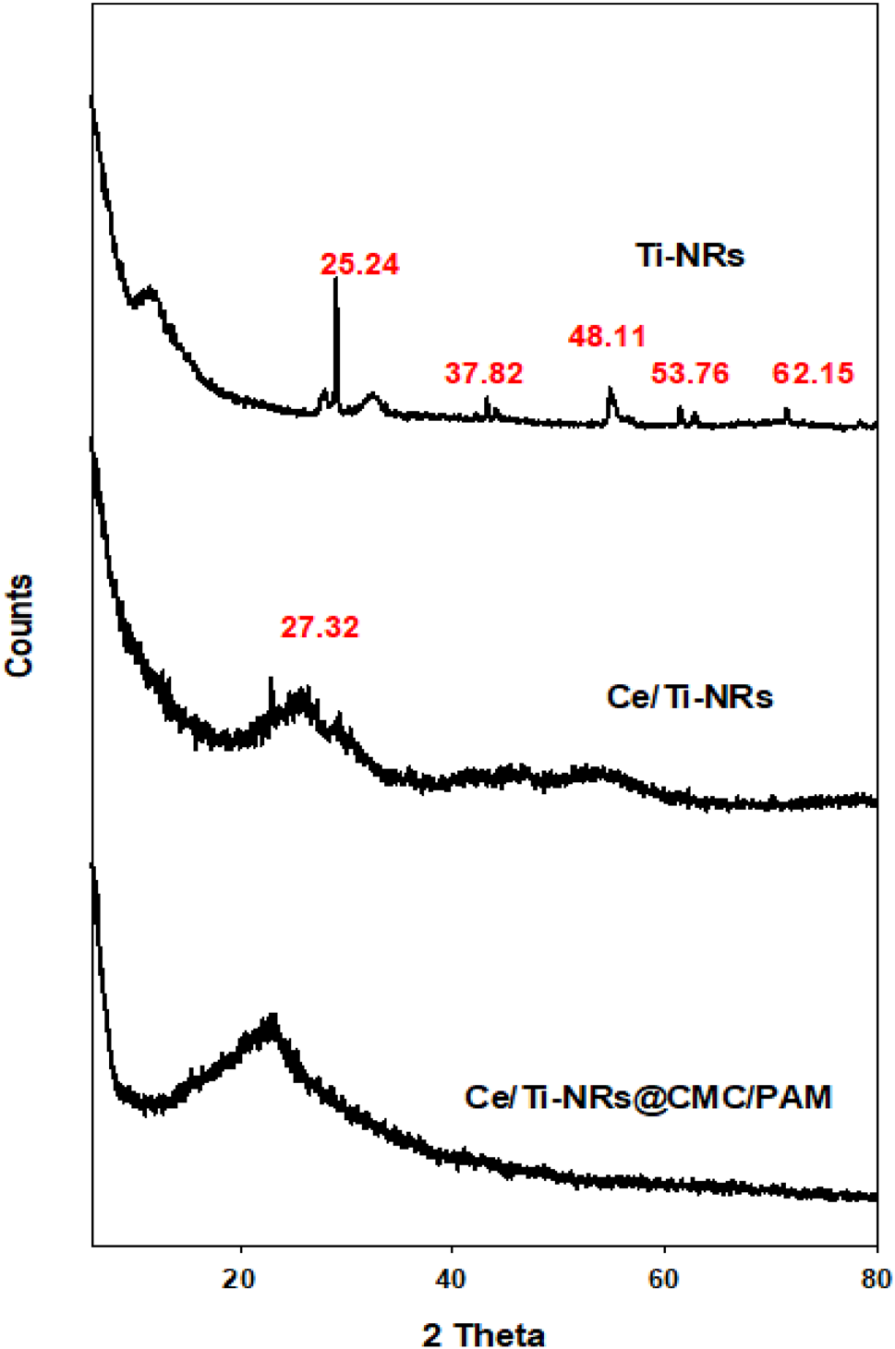
X-ray diffraction (XRD) patterns of Ti-NRs, Ce/Ti-NRs and Ce/Ti-NRs/CMC/PAM composite hydrogel.

Figure 3 demonstrates the XRD diffraction peaks of TiO_2_, TiO_2_-Ce and the Ce/Ti-NRs/CMC/PAM composite hydrogel. Two identifying peaks appear at 25.3° and 48.3°. They correspond to (101) and (200) planes referring to the anatase phase of titania nano-roads with intense peaks. Moreover, some other typical peaks appear at 37.82°, 53.76°, and 62.15°.They denote to (104), (105), and (204) planes respectively. Upon introducing cerium oxide, it has to mention that cerium does not replace titania but it is loaded onto it to modify its surface. Thus, the modified Ce/Ti-NRs did not show any characteristic XRD peak for any cerium oxide either (CeO_2_) or (Ce_2_O_3_). This condition may be due to the on the modified surface of TiO_2_ to be reveled^47^. The presence of both polymers’ polyacrylamide and carboxymethyl cellulose in the investigated hydrogel loaded with modified titania did not show characteristic XRD patterns except the first one of TiO_2_ appearing as a broad peak with some shifts to be at 22.36°.

Although TEM and SAED analyses clearly revealed CeO_2_ crystalline domains decorating the TiO_2_ nano-rods, the corresponding XRD patterns did not show distinct CeO_2_ peaks. This discrepancy is attributed to the low loading of Ce/Ti-NRs, the nano-scale crystallinity of CeO_2_, and the masking effect of the amorphous hydrogel matrix, which together reduce the detect ability of CeO_2_ by XRD. TEM/SAED, being more sensitive to local crystalline order, confirmed the presence of CeO_2_ phases.

#### Surface morphology and EDX analysis

**Fig. 4:**
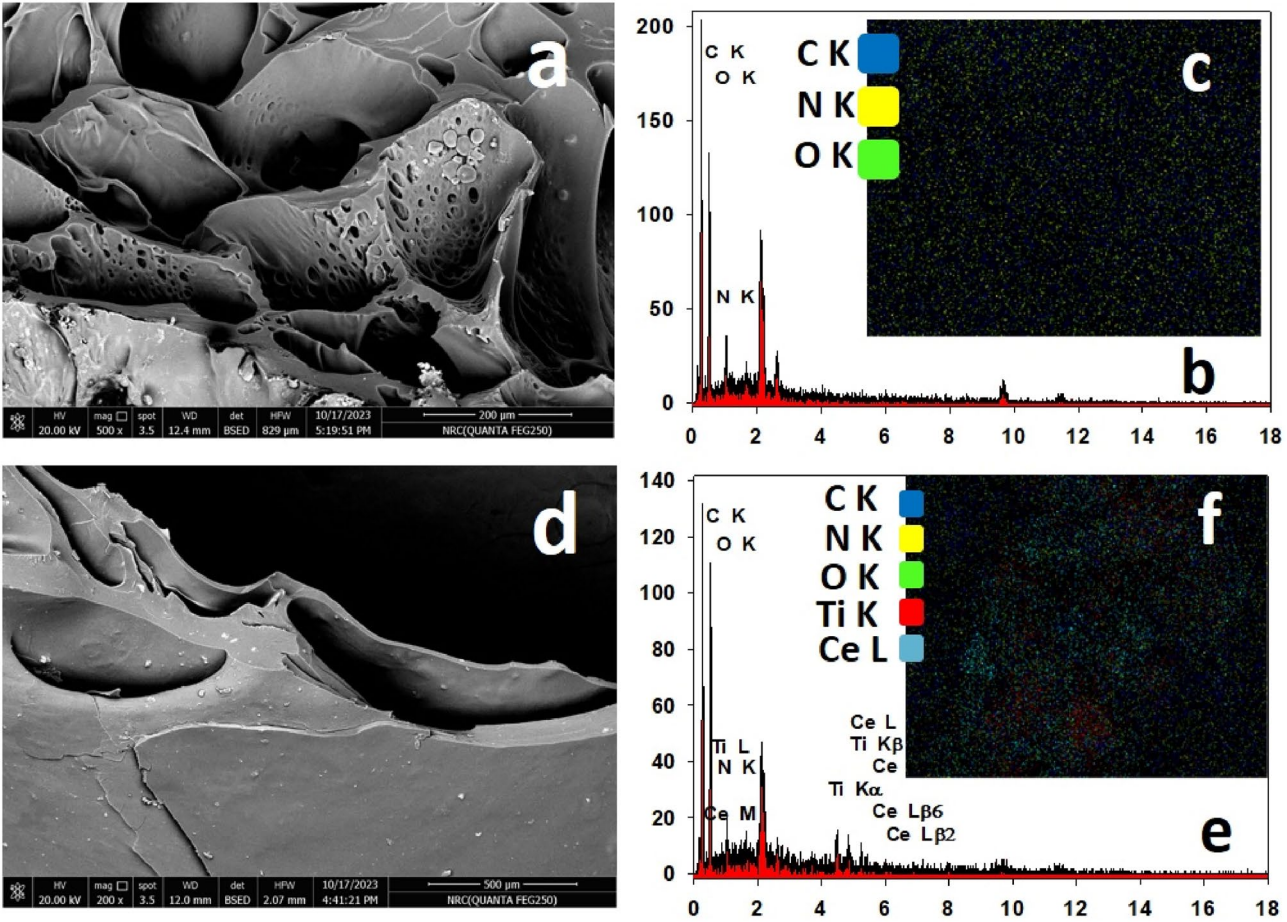
SEM images, EDX analysis, and elemental mapping of CMC/PAM hydrogel (**a–c**) and Ce/Ti-NRs/CMC/PAM composite hydrogel (**d**–**f**).

The surface morphology and elemental composition for CMC/PAM hydrogel and Ce/Ti-NRs/CMC/PAM composite hydrogel are represented in Fig. 4. A sponge-like structure for CMC/PAM hydrogel indicating a typical structure for polymeric hydrogels is shown in Fig. 4a. It illustrates a porous form with different pore sizes. In case of loading this hydrogel with Ce/Ti-NRs, some light spots appear across the whole surface of the modified hydrogel as depicted in Fig. 4d. The EDX analysis and the mapping of this hydrogel denoting to the presence of the main elements C, N and O of the utilized polymers represent by Fig. 4b,c. In addition, these techniques supported in emphasizing the presence of the metallic Ti and Ce nano-particles extended uniformly on the surface of the prepared hydrogel. Ti shows kβ and Lα at 4.5 and 0.4 keV respectively, while other peaks appear at 0.8 and 4.8 keV referring to Ce. This distinctive feature has been confirmed by the elemental mapping as displayed in Fig. 4e,f.

**Fig. 5:**
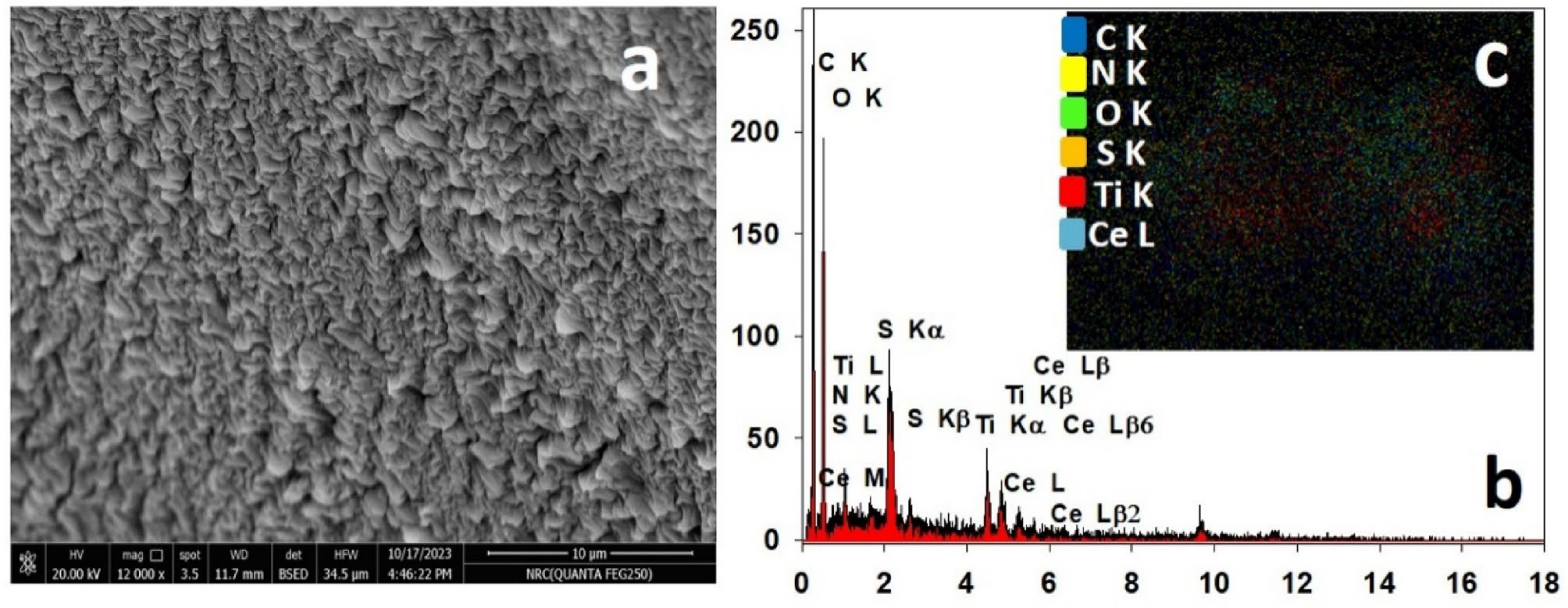
SEM images (**a**), EDX analysis (**b**), and elemental mapping (**c**) of Ce/Ti-NRs/CMC/PAM composite hydrogel after Cong red dye removal.

Figure 5 shows the SEM micrograph of Ce/Ti-NRs/CMC/PAM composite hydrogel after Congo red dye removal. The surface shows a kind of shrinkage in the surface of the hydrogel. It may due to the adsorption process leading to decreasing the size of the pores in this hydrogel. Moreover, a new characteristic EDX peak is noticed at 2.3 keV in Fig. 5b. It denotes to sulphur element which exists in the Congo red dye. The elemental mapping assists in verifying the appearance of (S) element in the used dye.

#### Brunauer–Emmett–Teller (BET) analysis

**Fig. 6:**
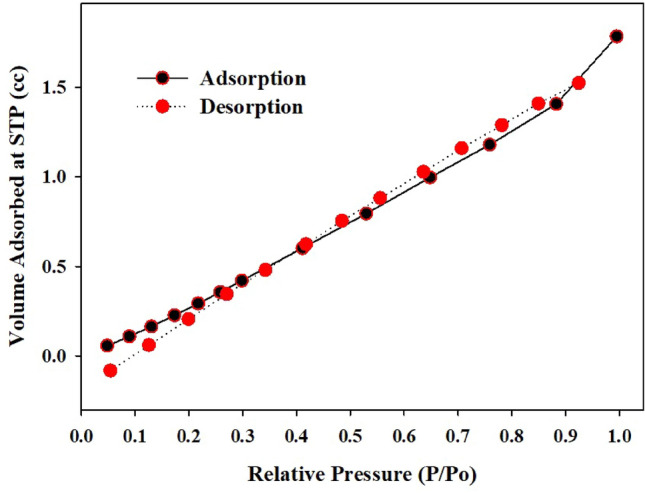
BET nitrogen adsorption plot of Ce/Ti-NRs/CMC/PAM composite hydrogel.

The Brunauer–Emmett–Teller (BET) analysis of the Ce/Ti-NRs/CMC/PAM composite hydrogel, Fig. 6**,** revealed a significant enhancement in the surface area of the hydrogel. The Ce/Ti-NRs/CMC/PAM composite hydrogel showed a surface area of 67.16 m^2^/g, an average pore radius of 1.92 nm, and a pore volume of 5.33 ± 0.052 cc/g. The nitrogen adsorption–desorption isotherms exhibited an indication of meso-porous structures. This increase in specific surface area can be attributed to the uniform dispersion of Ce/Ti-NRs within the hydrogel matrix, which introduced additional pore channels and prevented the collapse of the polymeric network. These isotherms exhibit a gradual and nearly linear increase in adsorbed volume with increasing relative pressure (P/P_0_). This behavior is characteristic of a Type II–like isotherm, indicating the presence of a predominantly non-microporous structure with accessible surface sites distributed throughout the hydrogel matrix. The absence of a sharp uptake at low relative pressure suggests that micro porosity is minimal. At the same time, adsorption primarily occurs through the formation of multi-layers on the internal surfaces of the polymer nano-rod composite. This trend reflects the open and hydrated nature of the CMC/PAM network, within which the Ce/Ti-NRs are effectively incorporated. A narrow hysteresis loop between the adsorption and desorption branches is observed over the intermediate to high relative pressure range. It points to the presence of meso-porous features within the hydrogel structure. This behavior suggests good structural integrity of the hydrogel after nanorod embedding, where the Ce/Ti-NRs do not collapse or severely obstruct the pore channels. Instead, they likely act as reinforcing fillers that stabilize the pore walls while maintaining smooth adsorption–desorption reversibility^48^. At higher relative pressures (P/P₀ > 0.7), the continued increase in nitrogen uptake can be attributed to capillary condensation within larger mesopores and macropore-like voids formed by the swollen hydrogel network. The close overlap of adsorption and desorption branches near saturation further indicates elastic pore behavior, which is typical for polymer-based hydrogels. Overall, the BET results confirm that embedding Ce/Ti-NRs preserves the porous architecture of the CMC/PAM hydrogel while providing additional surface exposure, a combination that is advantageous for applications requiring efficient mass transfer, adsorption, or surface-mediated reactions.

### Dye removal process

#### Effect of Ce/Ti-NRs/CMC/PAM composite hydrogel composition

In the first set of control experiments, to demonstrate the removal of the dye by adsorption or photo catalytic degradation, photolysis of the dye under visible light without a catalyst was performed, and it was found (< 4%). The adsorption equilibrium in darkness was ~ 38.5% removal, while subsequent irradiation increased removal to ~ 93%. These results demonstrate that although adsorption contributes to dye uptake, photo catalytic degradation by Ce/Ti-NRs is the dominant mechanism responsible for the high overall efficiency. Also, the adsorption experiment was conducted in the dark until equilibrium was reached. The maximum adsorption capacity was ~ 35 mg/g, corresponding to ~ 40% removal for the hydrogel. After attaining adsorption equilibrium, the system was exposed to visible light to separate the adsorption contribution from photocatalytic degradation. By comparing dye removal in darkness (adsorption only) versus under visible light (adsorption + photo catalysis), the photocatalysis added ~ 53% of the total removal beyond the adsorption equilibrium.

**Fig. 7:**
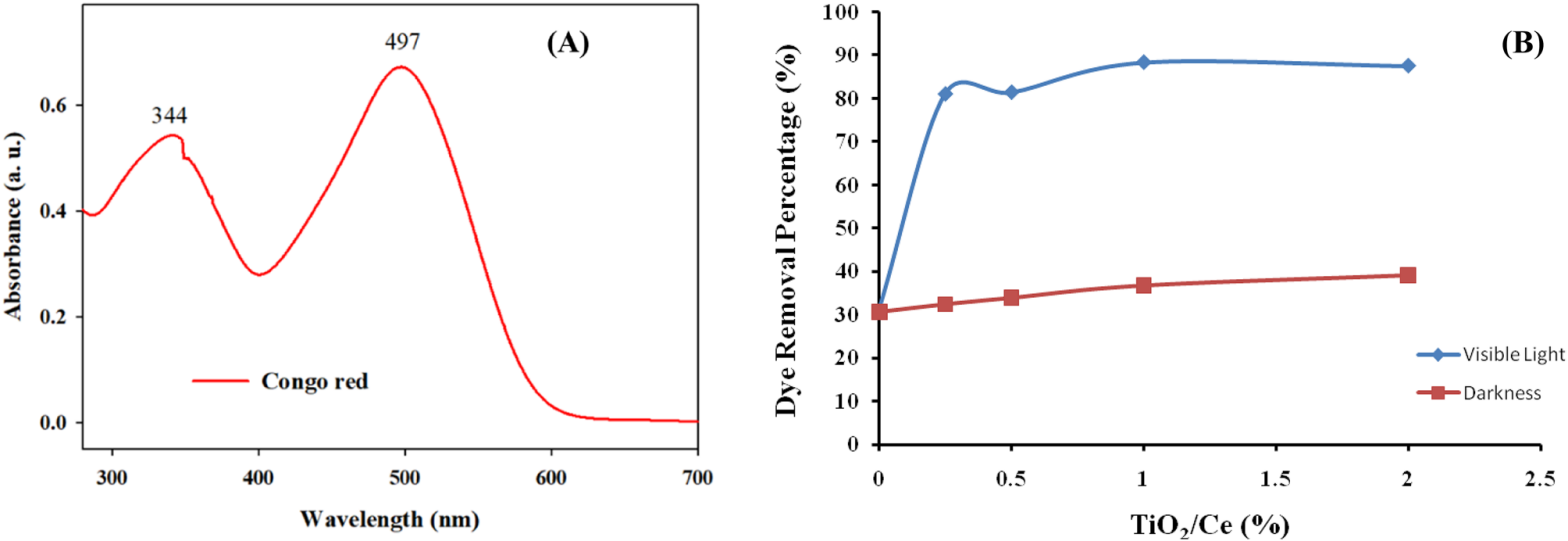
(**A**) UV–Vis spectrum of Congo red (15 ppm). (**B**) The effect of Ce/Ti-NRs/CMC/PAM composite hydrogel composition on the dye removal percentage in both darkness and visible light conditions.

The UV–Vis absorbance spectrum of CR dye at a concentration of 15 ppm (Fig. 7A) reveals two absorption bands, characteristic of its molecular structure and electronic transitions. The first band appears at approximately 344 nm, corresponding to the π → π* electronic transitions within the aromatic rings of the dye molecule^49^.

The second and more intense absorption peak is observed at 497 nm, which is attributed to n → π* transitions associated with the azo (-N = N-) chromophore and the auxochromic groups (such as sulfonic acid and amino functionalities) that enhance the dye’s visible light absorption^50^. The high absorbance at 497 nm makes this wavelength suitable for quantitative analysis of CR concentration via spectro-photometry. Figure 7B shows the effect of Ce/Ti-NRs/CMC/PAM composite hydrogel composition on the dye removal percentage in both darkness and visible light conditions, where the Ce/Ti-NRs content was varied from (0.25 to 2 wt. %). In the dark condition, the adsorption mechanism is the dominant one in the removal of the dye. It is clear that the addition of the Ce/Ti-NRs has a linear positive effect. The dye removal percentage increased linearly from 30% using the PAC1 sample (0% Ce/Ti-NRs) to reach 38.5% dye removal percentage using the PAC5 sample (2.0% Ce/Ti-NRs). On the other hand, the dye removal percentage under the visible light condition almost increased by 2.7 folds to 2.94 folds using PAC2 and PAC5 samples where the photo catalytic degradation effect of the Ce/Ti-NRs is shown up in addition simultaneously to the adsorption effect of the CMC/PAM crosslinked hydrogel backbone component to reach maximum dye removal percentage around 88.0%. PAC5 sample was selected for the dye removal study under different conditions.

#### Effect of contact time

**Fig. 8:**
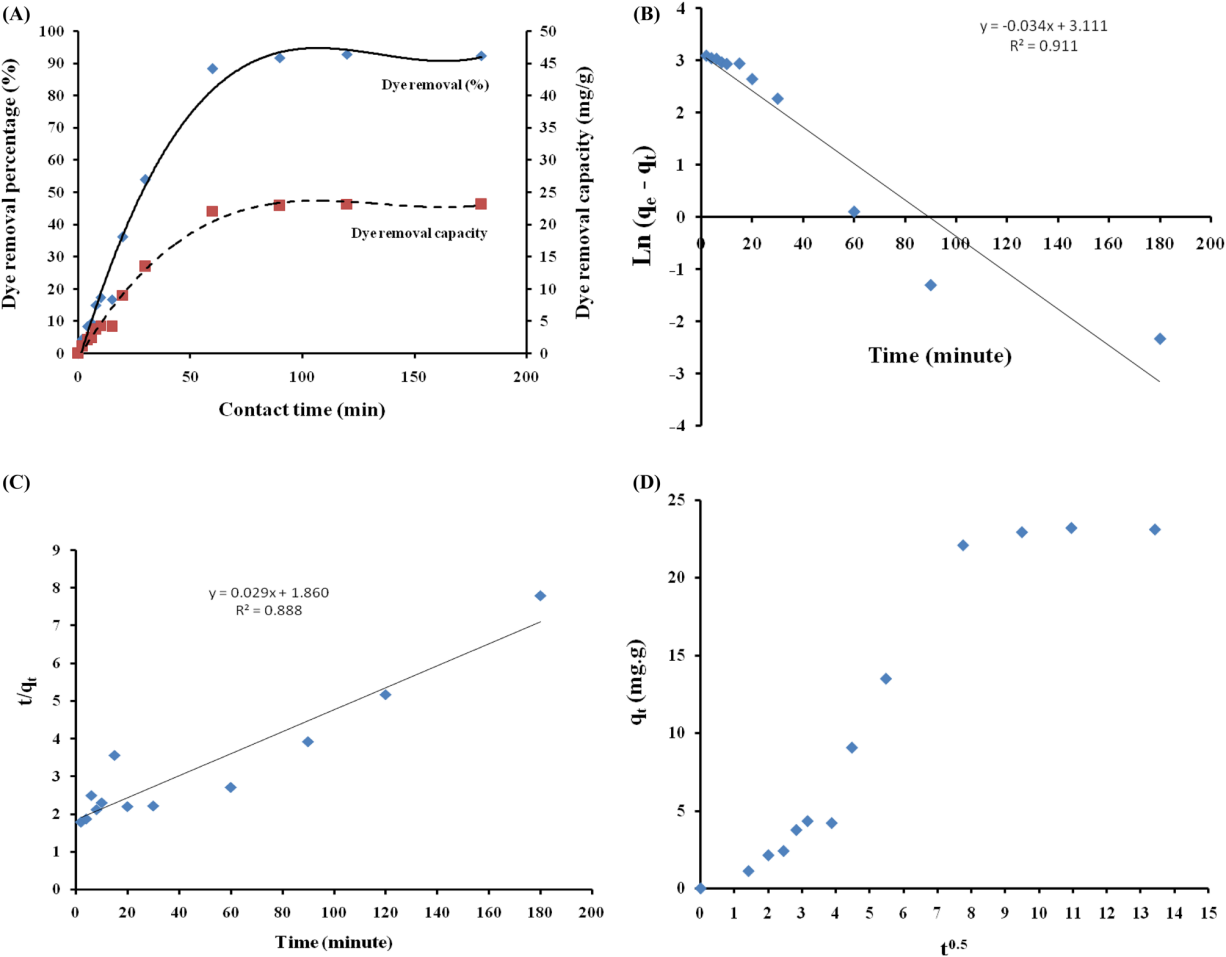
(**A**) The effect of contact time on the dye removal percentage under visible light conditions. (**B**) The pseudo first-order model adsorption kinetics model. (**C**) The pseudo second-order model adsorption kinetics model. (**D**) The intra-particle diffusion kinetic model.

The impact of the contact time under visible conditions on the dye removal percentage has been investigated in the duration of 180 min (Fig. 8A). As shown in Fig. 7, the cellulose-based hydrogel matrix has a contribution as an adsorbent in the dye removal process in addition to the photo degradation act of the nano-cerium titanate component. It is better to born in mind that processes, adsorption and photo degradation, are carried out simultaneously. The adsorbed dye molecules by the cellulose-based hydrogel are simultaneously photo degraded by the nano-cerium titanate component. From this Figure, it is obvious that a fast linear dye removal process has been recognized during the first 60 min where 88% of the dye has been removed. Then after 90 min, the dye removal percentage started leveling off and almost reached the equilibrium state with 92% dye removal percentage. No further dye removal has been noticed upon prolonging the contact time up to 180 min. Based on the obtained results, 90 min contact time was selected in the coming investigations. This behavior is related to the dye concentration gradient between the liquid phase and the solid composite phase. The difference between dye concentrations in the liquid phase and the solid composite phase is high. This feature acted as the driving force for the dye molecules to transfer from the liquid phase to the solid composite phase where simultaneously adsorbed and degraded. The adsorption capacities of the CMC/PAM hydrogel working in the same direction through opposite charges interaction with dye anions facilitate positioning dye molecules near the active sites of Ce/Ti-NRs, where hydroxyl radicals are produced. Moreover, holes and electrons efficiently interact with dye species adsorbed on the TiO_2_ catalyst surface to facilitate efficient dye degradation^31^. The dye removal capacity has shown the same trend reaching maximum value around 23 mg/g.

Kinetic studies for the removal (adsorption) of various pollutants, such as synthetic dyes and heavy metal ions, are essential because they provide information on the time required to reach adsorption equilibrium, the rate of adsorption, and the concentration of adsorbate in each phase after equilibrium has been reached^51^.To understand more clearly, the adsorption of CR dye onto Ce/Ti-NRs/CMC/PAM composite hydrogel, the kinetic behavior was studied using pseudo-first order (PFO) and pseudo-second order (PSO) as well as intra-particle diffusion (IPD) kinetics models. The linear equation for the model of pseudo-first order is^52^;

3 $$\ln \left( {q_{e} - q_{t} } \right) = \ln q_{e} - K_{1} t$$

where q_e_ is the adsorption amount of dye in mg/g at equilibrium**,** q_t_ (mg/g) is the amount of CR dye adsorbed at time t and K_1_ (min^-1^) is the first order kinetic model’s rate constant.

Table 2 summaries the values of K_1_, q_e_, and the correlation coefficient (R^2^) calculated from linear plots of ln (q_e_ − q_t_) versus t (Fig. 8B).

4 $$\frac{t}{{q_{t} }} = \frac{1}{{k_{2} q_{e}^{2} }} + \frac{t}{{q_{e} }}$$

**Table 2:** Removal (Adsorption-Degradation) kinetics and diffusion mechanism for removal of CR dye.

q_e-exp_(mg/g)	First-order kinetic parameter(PFO)			Second-order kinetic parameter(PSO)		
	K_1_ (min^-1^)	q_e-cal_ (mg/g)	R^2^	K_2_ (g/mg.min)	q_e-cal_(mg/g)	R
23.193	−0.034	22.44	0.911	1.86	34.78	0.888

Intra-particle diffusion(IPD)						
Adsorption			Saturation			
C	K	R^2^	C	K	R^2^	
−2.15	2.37	0.844	21.07	0.168	0.633	

K_2_ is the rate constant of pseudo second-order kinetic model’s adsorption (Fig. 8C).

The linear equation for intra-partical diffusion model is^53^;

5 $$qt = k_{p} t^{0.5} + c$$

K_p_ is the rate constant of the intra-particle diffusion kinetic model’s (mg g^-1^/min).Its value can be found from the slope of qt versus t^0.5^. C (mg g^-1^) is the intercept giving an indication of the boundary-layer thickness (Fig. 8D).The calculated parameters from the slope and intercept of the linear plots of the pseudo-first order, pseudo-second order, and intra-particle diffusion models are shown in Fig. 8B–D and tabulated in Table 2. It demonstrates that R^2^ of the pseudo-second order model (R^2^ = 0.888) is lesser than R^2^ of the pseudo-first order model (R^2^ = 0.911) for CR dye. Also, the calculated equilibrium capacity values (q_e-cal_ = 22.44 mg/g) form the pseudo-first order were found to be closer to the corresponding experimental values (q_e-exp_ = 23.193 mg/g) than those of pseudo-second order model (q_e-cal_ = 34.78 mg/g).They indicate that the pseudo-first order kinetic model can explain the kinetic of the removal (adsorption-degradation) of CR dye well using Ce/Ti-NRs/CMC/PAM composite hydrogel. The two-stage removal (adsorption-degradation) of CR dye using Ce/Ti-NRs/CMC/PAM composite hydrogel was disclosed by plotting q_t_ versus t^0.5^ as shown in Fig. 8D. The first stage may be attributed to the diffusion of CR molecules from the bulk to the exterior surface of the Ce/Ti-NRs/CMC/PAM composite hydrogel. The second stage, equilibrium has been attained. The linear plot of the intra-particle model (Table 2) revealed that the straight line has nonzero intercept values. It demonstrates that the removal (adsorption-degradation) of CR dye on Ce/Ti-NRs/CMC/PAM composite hydrogel is likely to be complex and involve both film diffusion (boundary layer diffusion) and intra-particle diffusion^54^.

#### Effect of dye concentration

**Fig. 9:**
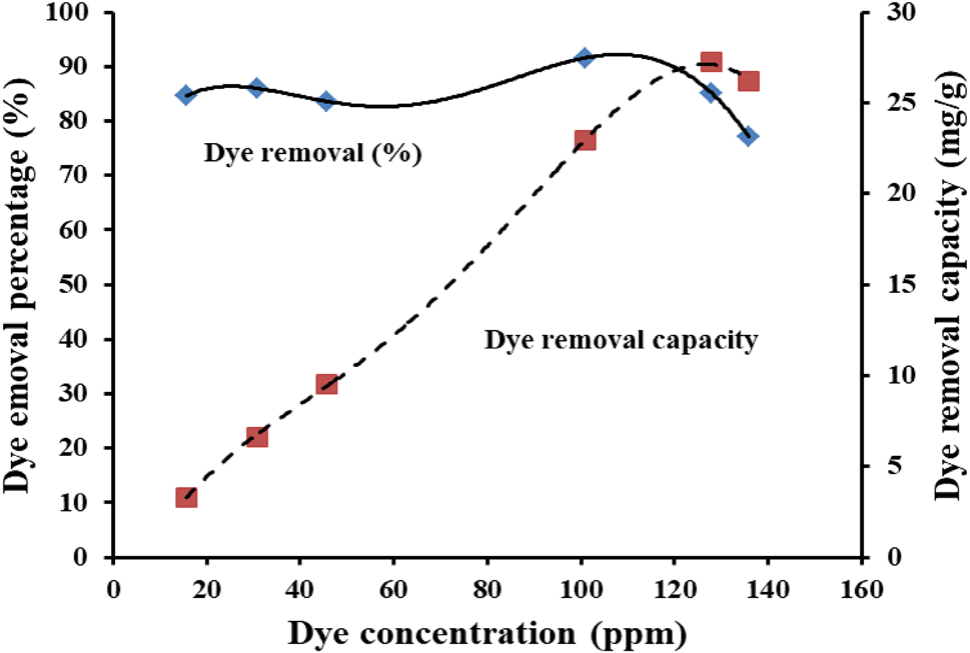
The effect of dye concentration on its removal percentage under visible light conditions.

Increasing the dye concentration of Congo red on the capability of the Ce/Ti-NRs/CMC/PAM composite hydrogel to remove the dye has been monitored under visible conditions as shown in Fig. 9. There are two significant observations can be detected. The first one is a slight maximum removal percentage, 91.6%, treating 100 ppm dye solution concentration. The second one is a decline of the removal percentage to 77.11% when treating 135 ppm of dye solution. Otherwise, the dye removal percentages fall in the range of 85% upon treating other dye concentrations. Two factors can explain the dye removal behavior. They are the dye concentration gradient between the dye solution and Ce/Ti-NRs/CMC/PAM composite hydrogel and the traffic resulted from the transportation of the photo-degraded dye fragments in the reverse direction. On the other hand, the dye removal capacity behavior has shown a linear increment with the dye concentration up to 128–135 ppm. It reached maximum value (26.20 mg/g-27.23 mg/g) and almost stabilized. The simultaneous adsorption-photo degradation steps make the adsorption capacities of the Ce/Ti-NRs/CMC/PAM composite hydrogel facilitate CR dye degradation by positioning CR dye molecules near the active sites of Ce/Ti-NRs, where hydroxyl radicals are produced. Moreover, holes and electrons efficiently interact with CR dye species adsorbed on the Ce/Ti-NRs catalyst surface to facilitate efficient CR dye degradation^31^.

#### Effect of the pH of the dye solution

**Fig. 10:**
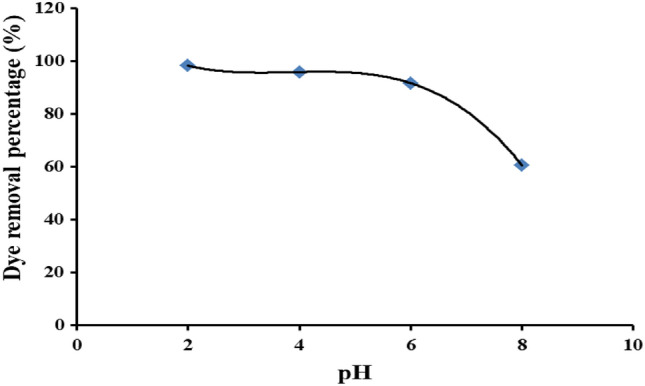
The effect of the pH of the dye solution on the dye removal percentage under visible light conditions.

The impact of the pH of the dye solution on the dye removal percentage is shown in Fig. 10 linear decrement of the dye removal percentage from 98.2 to 91.6% has been recognized in the pH range from 2.0 to 6.0. A sharp decline to 60.5% has been detected with increasing pH up to 8.0. Many factors could affect individually or synergistically the dye removal percentage. They can be summarized as follow:

1. The impact of the pH of the dye solution on the swelling of Ce/Ti-NRs/CMC/PAM composite hydrogel has directly affected the delivery of the dye solution to the interior part of the hydrogel.
2. The ionization of the functional groups influences both the hydrogel and the dye molecules.
3. The traffic of the dye photo degradation products is monitored from the hydrogel interior to the dye solution phase; against the dye molecules from the dye solution phase to the interior of the hydrogel.
4. Opposite charges show interaction between the dye photo degradation products and the hydrogel matrix.

The main advantage of the obtained result could be concluded in the high removal of the dye (over 90%) in a wide range of working pH dye solution (2.0 to 6.0). 

#### Effect of dye solution temperature

**Fig. 11:**
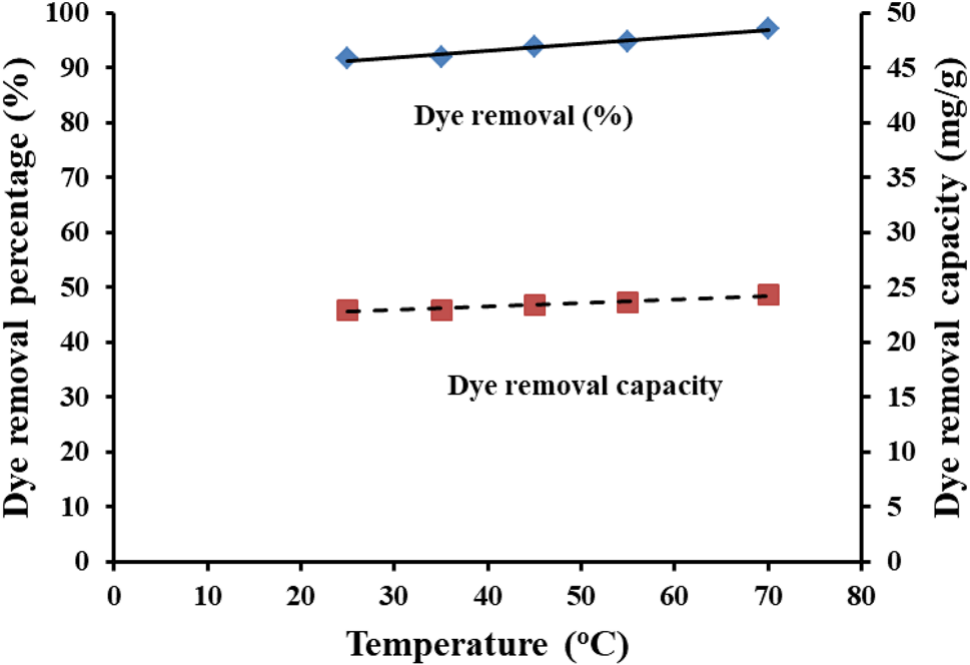
The effect of the dye solution temperature on the dye removal percentage and capacity under visible light conditions.

The influence of the working temperature on the dye removal percentage and capacity was investigated as shown in Fig. 11. A very slight effect of raising the working temperature from 25 to 70^O^C was depicted where the dye removal percentage and capacity linearly increased from 91.6 to 97.1% and 22.92 to 24.275 mg/g respectively as displayed in Fig. 11. This behavior can be explained by the results extracted from BET analysis; high surface area 67.16 m^2^/g, an average pore radius of 1.92 nm, and a pore volume of 5.33 ± 0.052 cc/g. The nitrogen adsorption–desorption isotherms exhibited an indication of meso-porous structures. This increase in specific surface area can be attributed to the uniform dispersion of Ce/Ti-NRs within the hydrogel matrix, which introduced additional pore channels and prevented the collapse of the polymeric network. This behavior is an advantage of the used Ce/Ti-NRs/CMC/PAM composite hydrogel matrix where no addition energy is required to obtain high dye removal percentage and consequently reduces the total cost of the process.

#### Effect of the dye solution stirring rate

**Fig. 12:**
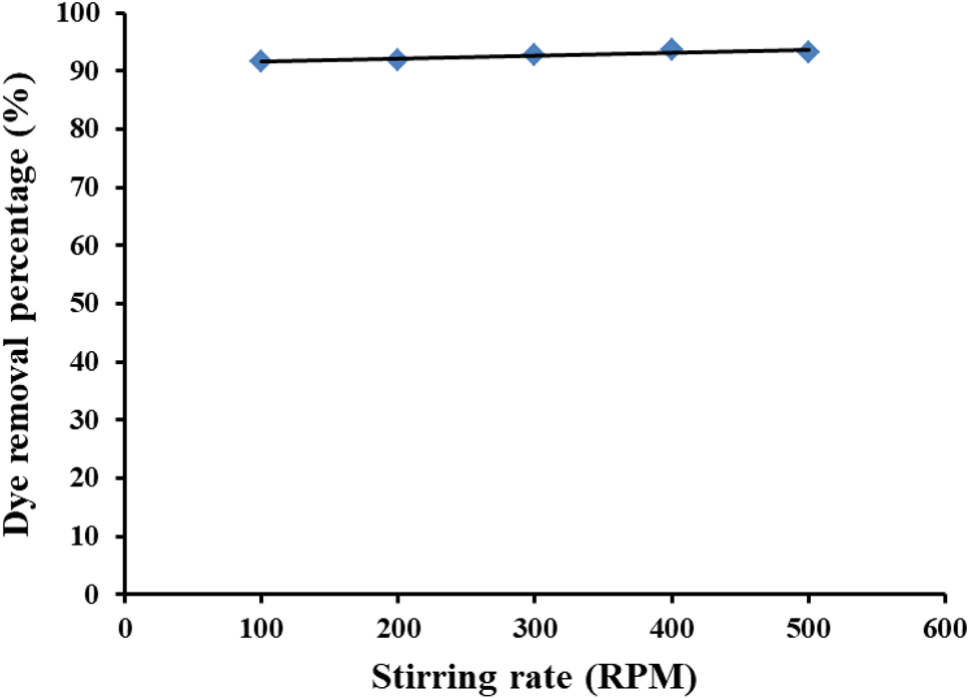
The effect of the dye solution stirring rate on the dye removal percentage under visible light conditions.

The accessibility of both adsorption and photo catalytic degradation center to the dye molecules is an important and determined factor in the dye removal process. From Fig. 12, it is clear that increasing the stirring rate has no significant effect on the dye removal percentage. Such behavior may be explained by the high surface area 67.16 m^2^/g, an average pore radius of 1.92 nm, and a pore volume of 5.33 ± 0.052 cc/g where the simultaneous adsorption-photo catalytic processes are mainly takes place. This behavior is advantageous as the used Ce/Ti-NRs/CMC/PAM composite hydrogel expresses that no additional energy is required to obtain high dye removal percentage and consequently reduces the total cost of the process.

#### Effect of adsorbent dose

**Fig. 13:**
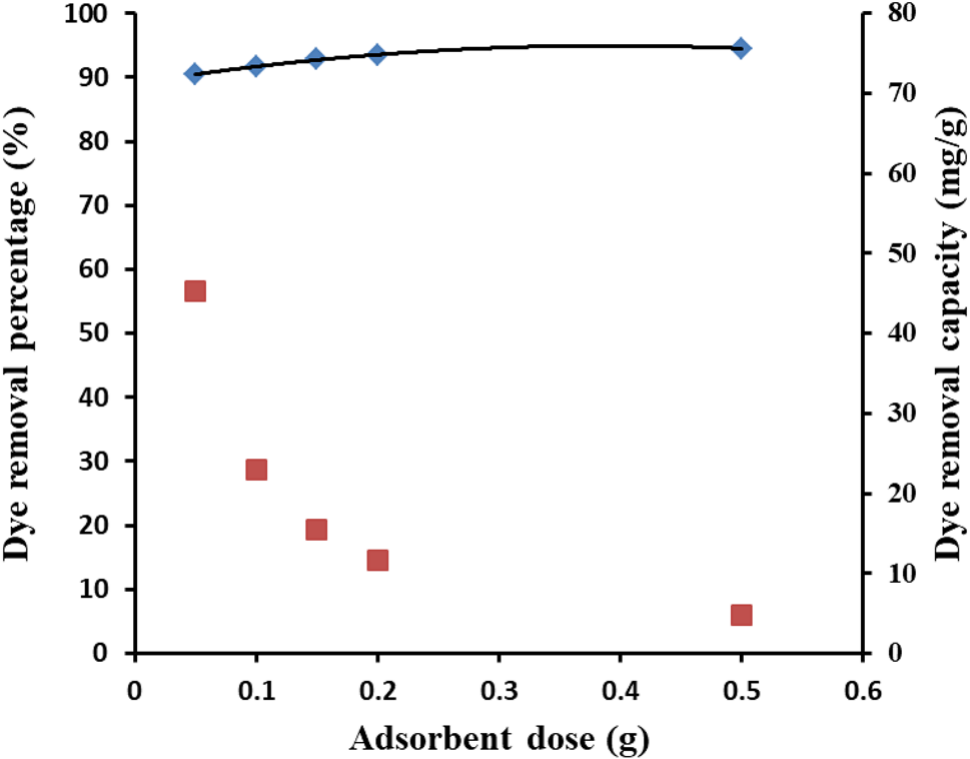
The effect of the Ce/Ti-NRs/CMC/PAM composite hydrogel adsorbent dose on the dye removal percentage and capacity under visible light conditions.

The impact of increasing the used Ce/Ti-NRs/CMC/PAM composite hydrogel dose in the removal of the Congo red from dye solution is presented in Fig. 13. The variation of the composite hydrogel dose from 0.05 to 0.5 g has a non-significant effect on the dye removal percentage which slightly increased from 90.5 to 94.5%. Such behavior indicates the dye concentration is a determining effect, from one side. The high dye removal capacity of the developed Ce/Ti-NRs/CMC/PAM composite hydrogel increased proportionally, from 4.725 to 45.25 mg/g, with decreasing the hydrogel dose from 0.5 to 0.05 g.

### Comparison of CR dye photo catalytic degradation

For commercializing any photo catalytic system, the efficiency of the system should be compared with the available literature. In the present work, direct sunlight has been utilized as an irradiation source for degradation of CR dye. Therefore, we compared this study with the earlier reported work in Table 3^55–58^. Thomas et al. ^55^ synthesized hydrogel of barium ion crosslinked alginate/carboxymethyl cellulose (CMC) with encapsulating TiO_2_-NPs successfully using dissipative convective method followed by freeze-drying procedure. The Ba/Alg/CMC/TiO_2_ hydrogel confirms that photo catalyst is highly photo stable where CR degradation (%) reached 91.5% after 240 min. S. Ramadhani & H. Helmiyati investigated alginate/CMC/ZnO nano-composites, where 90.12% degradation was obtained after only 110 min^56^. Incorporating Sr and GO into Sr/Alg/CMC/GO/TiO_2_ composite successfully degraded 98% of CR dye after 240 min^57^. Integration of carboxymethyl cellulose isolated from oil palm into bismuth ferrite as photo catalyst for CR degradation by Md Azman et al.^58^ obtained 95.49% degradation after 180 min. This work successively degraded 91.68% of CR dye only after 90 min.

**Table 3:** Degradation percentages of Congo red dye using different photo catalysts under direct sunlight.

Catalyst	Time (min.)	Degradation (%)	Reference
Ba/Alg/CMC/TiO_2_	240	91.5	^55^
Alginate/CMC/ZnO nanocomposites	110	90.12	^56^
Sr/Alg/CMC/GO/TiO_2_	240	98	^57^
CCMC/BFO	180	95.49	^58^
Ce/Ti-NRs/CMC/PAM composite hydrogel	90	91.68	This work

## Conclusion

The developed novel Ce/Ti-NRs/CMC/PAM composite hydrogel shows high degradation capacity by simultaneously combining the adsorption technique through the PAM/CMC crosslinked hydrogel and the photo catalytic degradation activity by the included cerium titanate nano-rods. The synthesized Ce/Ti-NRs, CMC/PAM, and Ce/Ti-NRs/CMC/PAM composite hydrogel have been characterized using different analysis techniques namely; FTIR, XRD, TEM, SAED, SEM, EDAX, and BET. The photo degradation process of the CR dye was investigated under different experimental conditions namely; dye concentration, contact time, Dye solution pH and temperature, adsorbent dose, and finally agitation rate. In total, 91.68% of CR dye was removed (adsorbed and photo degraded) only after 90 min which was found superior to other relevant published results.

The results demonstrate high efficiency in Congo red degradation; systematic analyses of recyclability, structural stability, and Ce/Ti leaching were not performed and remain limitations of the present work. Given the dual crosslinking strategy employed, we anticipate good immobilization and reusability, but these aspects will be addressed in future studies to fully validate the composite hydrogel practical applicability. Moreover, the surface area, porosity, pore volume effect on the kinetic of the dye simultaneously adsorption-degradation process is critical issue especially when born in mind that the Ce/Ti-NRs/CMC/PAM composite hydrogel has high surface area 67.16 m^2^/g, an average pore radius of 1.92 nm, and a pore volume of 5.33 ± 0.052 cc/g despite its micro-particle size; 400–600 microns. It is expected to have higher surface area using smaller Ce/Ti-NRs/CMC/PAM composite hydrogel particles and consequently higher rate of dye removal leading to the reduction of the treatment time and cost.

## Data Availability

All data generated or analyzed during this study are included in this published article.
